# Unraveling the relationship between serum parathyroid hormone levels and trabecular bone score: a cross-sectional study

**DOI:** 10.1038/s41598-024-63979-9

**Published:** 2024-06-06

**Authors:** Tingxiao Zhao, Yanlei Li, Jinlong Tian, Yao Kang, Jiongnan Xu, Haiyu Shao, Jinlei Zhou, Chen Xia, Yongguang Wang, Jun Zhang

**Affiliations:** 1 Center for Plastic & Reconstructive Surgery, Department of Orthopedics, Zhejiang Provincial People’s Hospital (Affiliated People’s Hospital, Hangzhou Medical College), Hangzhou, Zhejiang China; 2 Department of Orthopedics, Linping Hospital of Integrated Traditional Chinese and Western Medicine, Linping District, No.60, Baojian Road, Hangzhou, 311199 Zhejiang China; 3 Department of Orthopedics, Zhejiang Provincial People’s Hospital Bijie Hospital, Guanghui Road 112#, Bijie, Guizhou, 551700 China

**Keywords:** Endocrine system and metabolic diseases, Patient education, Osteoporosis

## Abstract

The TBS is a new method for clinicians to assess the bone quality. It is directly related to the mechanical strength of bone and helps predict fracture risk. The present analysis aimed to investigate the associations between serum PTH levels and TBS by analyzing data from the National Health and Nutrition Examination Survey (NHANES). A total of 3516 participants from the NHANES 2005–2006 were included in this cross-sectional study. The independent variable was serum PTH, and the outcome variable was TBS. The associations of serum PTH levels with TBS were examined using multivariable linear regression models. After adjusting for covariates, there was a negative association between serum PTH level and TBS (β = − 0.0034; 95% confidence interval, − 0.0050 to − 0.0017). However, in the subgroup analysis stratified by gender, race, and age, this association became negative only in Non-Hispanic White (β =  − 0.0047, 95% CI:  −  0.0071 to  −  0.0048) and young people (age < 60) (β = − 0.0036, 95% CI: − 0.0057, − 0.0016), regardless of gender. In addition, the association of serum PTH with TBS was an U-shaped curve, with a point of inflection at 6.71 pmol/L. This study showed that serum PTH level was negatively associated with TBS. Maintaining PTH levels in a lower reasonable clinical range may be beneficial to bone health, especially for young non-Hispanic white.

## Introduction

Osteoporosis, the most common skeletal disorder of all, is a combination of bone mineral density (BMD) (i.e., quantity) and altered bone quality (i.e., quality), resulting in decreased bone strength with an increased risk of fractures at various sites like hip, wrist, and vertebrae^1–3^. In the United States, an estimated 10.2 million people aged 50 years and above have osteoporosis, and an additional 43.3 million have low bone mass^4^. In addition, there are more than 2 million fractures related to osteoporosis annually, which generates a cost of nearly USD 13.7–20.3 billion^5^. The incidence of fragility fractures is increasing rapidly due to the increasing population of older adults. Among fragility fractures, hip fractures are associated with the greatest disability and mortality^6^. Thus, osteoporosis as a systemic skeletal disease has a significant impact on the associated morbidity, mortality, health-care expenses, and burden of disease.

The BMD measured by dual X-ray bone densitometer (DXA) is considered to be the gold standard for the diagnosis of osteoporosis in the absence of definite brittle fracture^7^. Bone mineral density (BMD) is an important indicator of bone strength and fracture risk, but it does not fully reveal the status of bone microstructure. Relevant studies^8^ have found that about 45% of fractured and non-fractured individuals overlap in their BMD test results, suggesting that relying on BMD alone to predict fracture risk is not ideal. Trabecular bone score (TBS) is a novel gray-texture measurement method, that derived from the lumbar spine DXA imaging^9^. It uses experimental variograms of 2D projection images, quantifying variation in grey-level texture from 1 pixel to the adjacent pixels, and has been widely used in clinical evaluation of bone quality in recent years^10^^.^ TBS reflects the bone microstructure index, which is directly related to the mechanical strength of bone^11^. Higher values of TBS indicate a better microarchitecture, whereas lower values indicate a degraded microarchitecture. In addition, TBS's prediction of fracture risk has been widely reported in cross-sectional, prospective and longitudinal studies, and has been endorsed by medical societies of bone field (IOF, the European Society for Clinical and Economic Aspects of Osteoporosis and Osteoarthritis, and the ISCD)^12,13^.

Parathyroid hormone (PTH) is a polypeptide hormone secreted by the chief cells of the parathyroid gland, and it is composed of 84 amino acids^14^. Its analogues are currently approved as osteoanabolic drugs for the treatment of osteoporosis, such as teriparatide, which is the active fragment of the amino-terminal 1-34 of PTH^15^. PTH combines with parathyroid hormone type 1 receptor (PrlHlR) in bone tissue to play a role in regulating bone metabolism. However, PTH has a two-way regulatory effect of promoting bone formation in small dose and intermittent, and promoting bone resorption in large dose and continuous^16^.

PTH has a well-known role in mineral homeostasis and bone metabolism. However, to our knowledge, no study has yet analyzed the relationship between serum PTH levels and TBS in the general population. This analysis aimed to explore the relationship between the two by examining data from the National Health and Nutrition Examination Survey (NHANES).

## Materials and methods

### Statement of ethics

NHANES is a cross-sectional study conducted on the American population, aimed at assessing the health and nutritional status of residents in the United States by collecting information on household demographics, health, and nutrition. Detailed data about it can be found on the internet and has been approved by the National Center for Health Statistics (NCHS) Ethics Review Board.

### Study population

The health and nutritional status of adults and children in the USA and is administered by the Centers for Disease Control and Prevention (CDC). NHANES, a representative survey of the USA national population, utilizes a complex, multi-stage probability sampling design to offer extensive information on the nutrition and health of the USA population^17^. Its goal is to help improve the current situation and prevent potential future health problems. This nationwide survey is conducted every 2 years.

Our study was based on data from the NHANES between 2005 and 2006.

**Figure 1:**
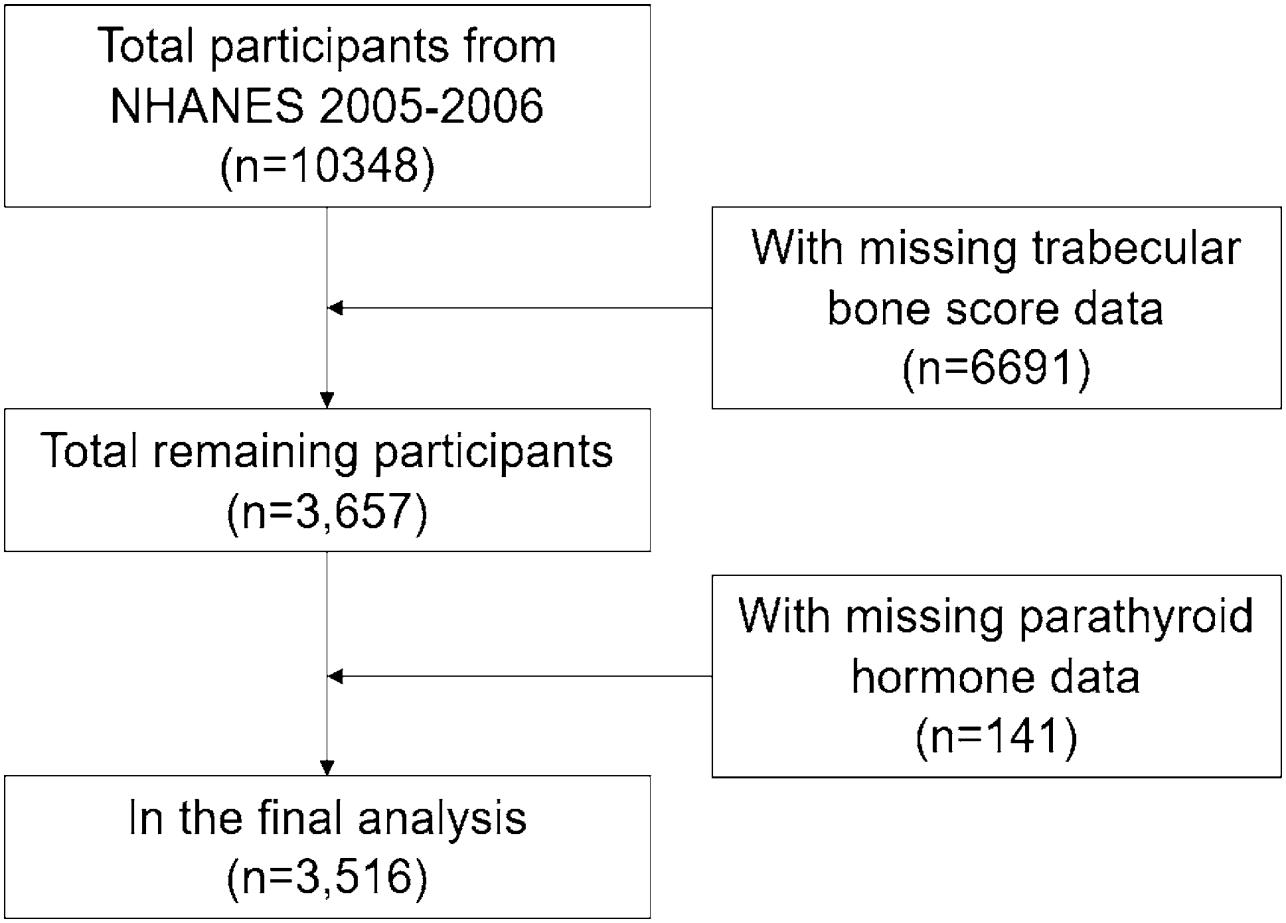
Flowchart of sample selection.

We included 3516 individual (1830 men and 1686 women). We excluded individuals with missing TBS (n = 6691) or serum PTH (n = 141) data. After selection, 3516 individual were included in our final analysis (Fig. 1).

### Variables

BMD and TBS were assessed using DXA at the lumbar spine (L1–L4). The DXA scans were performed using a Hologic QDR-4500A fan-beam densitometer (Hologic, Inc., Bedford, Massachusetts), and all participant scans were reviewed and analysed by experts at the University of California, San Francisco (UCSF). For individuals aged 20 and older, the TBS software (Med-Imap SA TBS Calculator version 2.1.0.2) was used to calculate trabecular bone scores for each lumbar vertebra and an overall TBS score for the total lumbar spine. This assessment is based on analyzing the variations in gray levels within the pixels of an anterior–posterior lumbar spine DXA scan. To ensure the accuracy of the data, the entire DXA scanning process follows strict steps and implementation standards, and more detailed information is documented in the Body Composition Measurement Manual on the NHANES website (http://www.cdc.gov/nchs/nhanes.htm). The measurement of serum parathyroid hormone was carried out using the Elecsys 1010 fully automatic analyzer, employing the sandwich method as the measurement assay. The normal values for parathyroid hormone vary depending on the testing method used.The reference range for normal values in this study is 11-67 pg/mL (1.16–7.05 pmol/L). We chose these covariates based on their links to the outcomes of interest or a change in the effect estimate exceeding 10%. For covariates, continuous variables included age, Poverty income ratio (PIR), BMI, Total spine BMD, alkaline phosphatase, blood urea nitrogen, total cholesterol, total protein, serum uric acid, calcium, creatinine, phosphorus, high density lipoprotein cholesterol (HDL-C), low density lipoprotein-cholesterol (LDL-C), C-reactive protein (CRP), Vit D. Categorical variables included gender, race, alcohol, coronary heart disease, physical activity, cancer, smoking.

### Statistical analysis

We performed all statistical analyses by using R (http://www.R-project.org) and EmpowerStats (http://www.empowerstats.com), with statistical significance set at P < 0.05. All estimates were calculated by using sample weights following the analytical guideline edited by National Center for Health Statistics (NCHS).

Three models were executed to adjust for covariates: Model 1 was non-adjusted. Model 2 was adjusted for age, gender, ethnicity. Model 3 included all variables from Model 2 and was further adjusted for PIR, BMI, total spine BMD, alkaline phosphatase, blood urea nitrogen, total cholesterol, total protein, serum uric acid, calcium, creatinine, phosphorus, HDL-C, LDL-C, CRP, Vit D, alcohol, coronary heart disease, physical activity, cancer, smoking. We also compared between-group differences using weighted chi-square tests and regression analyses. In addition, the linear or nonlinear association between PTH and TBS was further explored by introducing a generalised additive model (GAM) and smoothed curve fitting in the complete adjusted model. The presence of a non-linear relationship was determined by the log-likelihood ratio. If a non-linear relationship is confirmed, we will use a segmented linear regression model and determine the turning point through a recursive algorithm.

### Ethics statement

According to local regulations and institutional requirements, this study did not necessitate ethical review and approval.

## Results

### Participant characteristics

A total of 3516 participants were included in our study, the detailed screening of study participants was presented in Fig. 1. The weighted characteristics of the participants subclassified based on PTH quartiles were demonstrated in Table 1.

**Table 1:** Characteristics of the study participants according to serum parathyroid hormone levels.

Characteristics		PTH levels (pmol/L)				P Value
		Q1 (0.70–3.41)	Q2 (3.41–4.51)	Q3 (4.51–5.83)	Q4 (5.83–54.01)	
Age		40.84 ± 14.53	45.59 ± 15.49	49.55 ± 16.22	52.68 ± 16.55	< 0.0001
Gender	Male	464 (56.59%)	498 (54.37%)	430 (50.00%)	438 (47.61%)	< 0.0001
	Female	356 (43.41%)	418 (45.63%)	430 (50.00%)	482 (52.39%)	
Race/ethnicity (%)						< 0.0001
	Non-Hispanic White	484 (59.02%)	471 (51.42%)	440 (51.16%)	415 (45.11%)	
	Non-Hispanic Black	141 (17.20%)	177 (19.32%)	175 (20.35%)	275 (29.89%)	
	Mexican American	149 (18.17%)	193 (21.07%)	188 (21.86%)	174 (18.91%)	
	Other Race	46 (5.61%)	75 (8.19%)	57 (6.63%)	56 (6.09%)	
PIR		2.73 ± 1.57	2.83 ± 1.69	2.75 ± 1.59	2.69 ± 1.57	0.426
BMI		26.86 ± 5.25	27.66 ± 5.33	28.67 ± 5.76	29.73 ± 6.26	< 0.001
Total spine BMD, gm/cm^2^		1.05 ± 0.14	1.04 ± 0.15	1.03 ± 0.15	1.03 ± 0.17	0.038
TBS		1.42 ± 0.13	1.39 ± 0.14	1.36 ± 0.14	1.33 ± 0.15	< 0.001
Alkaline phosphatase, mg/dl		66.45 ± 24.90	70.30 ± 24.35	70.44 ± 22.05	77.71 ± 31.85	< 0.001
Blood urea nitrogen, mg/dl		12.22 ± 4.61	12.45 ± 4.43	12.93 ± 4.96	14.33 ± 7.76	< 0.001
Total cholesterol, mg/dl		198.86 ± 41.92	198.93 ± 40.85	201.13 ± 45.36	197.89 ± 40.65	0.429
Total protein, mg/dl		7.15 ± 0.47	7.19 ± 0.50	7.13 ± 0.44	7.12 ± 0.47	0.014
Serum uric acid, mg/dl		5.20 ± 1.28	5.35 ± 1.34	5.43 ± 1.33	5.72 ± 1.55	< 0.001
Calcium, mg/dl		9.60 ± 0.33	9.53 ± 0.32	9.47 ± 0.31	9.42 ± 0.42	< 0.0001
Creatinine, mg/dl		0.92 ± 0.19	0.92 ± 0.21	0.93 ± 0.20	1.07 ± 0.78	< 0.0001
Phosphorus, mg/dl		3.88 ± 0.57	3.83 ± 0.55	3.76 ± 0.53	3.72 ± 0.58	< 0.0001
HDL-C, mg/dl		54.13 ± 16.67	53.34 ± 15.28	54.25 ± 16.19	54.50 ± 16.52	0.758
LDL-C, mg/dl		116.97 ± 37.46	116.86 ± 35.92	119.42 ± 38.99	114.72 ± 37.29	0.147
CRP, mg/dl		0.39 ± 1.07	0.41 ± 0.71	0.41 ± 0.69	0.50 ± 0.69	< 0.0001
Vit D, mg/dl		64.30 ± 21.16	58.30 ± 20.04	54.97 ± 18.53	50.21 ± 19.03	< 0.0001
Alcohol	Yes	98 (11.95%)	147 (16.05%)	147 (17.09%)	174 (18.91%)	< 0.0001
	No	68 (8.29%)	105 (11.46%)	120 (13.95%)	134 (14.57%)	
	Missing	654 (79.76%)	664 (72.49%)	593 (68.95%)	612 (66.52%)	
Coronary heart disease	Yes	17 (2.07%)	20 (2.18%)	37 (4.30%)	56 (6.09%)	< 0.0001
	No	800 (97.56%)	895 (97.71%)	820 (95.35%)	855 (92.93%)	
	Missing	3 (0.37%)	1 (0.11%)	3 (0.35%)	9 (0.98%)	
Physical activity	Yes	362 (44.15%)	391 (42.69%)	354 (41.16%)	383 (41.63%)	< 0.0001
	No	210 (25.61%)	196 (21.40%)	172 (20.00%)	149 (16.20%)	
	Missing	248 (30.24%)	329 (35.92%)	334 (38.84%)	388 (42.17%)	
Cancer	Yes	47 (5.73%)	74 (8.08%)	80 (9.30%)	95 (10.33%)	0.012
	No	773 (94.27%)	840 (91.70%)	780 (90.70%)	824 (89.57%)	
	Missing	0 (0.00%)	2 (0.22%)	0 (0.00%)	1 (0.11%)	
Smoking	Yes	467 (56.95%)	453 (49.45%)	401 (46.63%)	409 (44.46%)	< 0.0001
	No	351 (42.80%)	463 (50.55%)	459 (53.37%)	511 (55.54%)	
	Missing	2 (0.24%)	0 (0.00%)	0 (0.00%)	0 (0.00%)	

Data are expressed as weighted means ± SD or percentages (%).

PIR, poverty income ratio; BMI, body mass index; BMD, bone mineral density; TBS, trabecular bone score; HDL-C (high-density lipoprotein cholesterol), LDL-C (low density lipoprotein-cholesterol), CRP (C-reactive protein).

Q1: 0.70 pmol/L ≤ PTH levels ≤ 3.41 pmol/L; Q2: 3.41 pmol/L < PTH levels ≤ 4.51 pmol/L; Q3: 4.51 pmol/L < PTH levels ≤ 5.83 pmol/L; Q4: 5.83 pmol/L < PTH levels ≤ 54.01 pmol/L.

### Association between serum PTH levels and TBS

The relationship between the serum PTH level and TBS is presented in Table 2. In our analysis, we found a significant negative correlation between serum PTH levels and TBS. Even after adjusting for all covariates, this negative correlation still exists (− 0.0034; 95% CI, − 0.0050, − 0.0017; P < 0.001). To evaluate the robustness of results, PTH was treated as categorical variables (quartiles) for sensitivity analysis. The general trends were consistent in all models from the Q1 to Q4. In four distinct models, taking Q1 as a reference, differences were detected for TBS across PTH quartiles (all P for trend < 0.05) (Table 3).

**Table 2:** Association between the serum parathyroid hormone levels and the trabecular bone score.

Variable	Model 1	P value	Model 2	P value	Model 3	P value
PTH (pmol/L)	− 0.0124 (− 0.0142, − 0.0106)	< 0.000001	− 0.0362 (− 0.0515 0.0208)	< 0.000001	− 0.0034 (− 0.0050, − 0.0017)	0.000052

Data are presented as β coefficient (95% CI).

Adjusted covariates: Model 1 = non-adjusted; Model 2 = Age, Sex, Race; Model 3 = Model 2 + PIR, BMI, Total spine BMD, Alkaline phosphatase, Blood urea nitrogen, Total Cholesterol, Total protein, Serum uric acid, Calcium, Creatinine, Phosphorus, HDL-C, LDL-C, CRP, Vit D, Alcohol, Coronary heart disease, Physical activity, Cancer, Smoking.

Abbreviations: see Table 1.

**Table 3:** Association between serum parathyroid hormone levels and trabecular bone score.

	Model 1 β (95% CI)	Model 2 β (95% CI)	Model 3 β (95% CI)
PTH levels (pmol/L) (quartile)			
Q1 (0.70–3.41)	Reference	Reference	Reference
Q2 (3.41–4.51)	-0.0342 (-0.0465, -0.0219) < 0.000001	-0.0144 (-0.0255, -0.0034) 0.010446	-0.0183 (-0.0283, -0.0083) 0.000325
Q3 (4.51–5.83)	− 0.0585 (− 0.0713, − 0.0458) < 0.000001	− 0.0230 (− 0.0346, − 0.0114) 0.000107	− 0.0273 (− 0.0379, − 0.0167) < 0.000001
Q4 (5.83–3.41)	− 0.0926 (− 0.1056, − 0.0795) < 0.000001	− 0.0439 (− 0.0561, − 0.0318) < 0.000001	− 0.0372 (− 0.0485, − 0.0258) < 0.000001
P for trend	< 0.001	< 0.001	< 0.001

Data are presented as β coefficient (95% CI).

Adjusted covariates: Model 1 = non-adjusted; Model 2 = Age, Sex, Race; Model 3 = Model 2 + PIR, BMI, Total spine BMD, Alkaline phosphatase, Blood urea nitrogen, Total Cholesterol, Total protein, Serum uric acid, Calcium, Creatinine, Phosphorus, HDL-C, LDL-C, CRP, Vit D, Alcohol, Coronary heart disease, Physical activity, Cancer, Smoking.

Abbreviations: see Table 1.

### Sex differences in the relationship between serum PTH levels and TBS

A linear regression of the sex specific association between the serum PTH level and TBS is shown in Table 4. A significant association was observed in men (β = − 0.0107; 95% CI, − 0.0133, − 0.0081) and women (β = − 0.0143; 95% CI, − 0.0169, − 0.0117). Even after adjusting for all covariates, negative associations were still evident in men (β = − 0.0030; 95% CI, − 0.0051, − 0.0008) and women (β = − 0.0038; 95% CI, − 0.0061, − 0.0015; P < 0.05).

**Table 4:** Association between serum parathyroid hormone levels and trabecular bone score, stratified by gender and race.

	Model 1 β (95% CI)	Model 2 β (95% CI)	Model 3 β (95% CI)
Stratified by gender			
Male	− 0.0107 (− 0.0133, − 0.0081) < 0.000001	− 0.0039 (− 0.0064, − 0.0014) 0.002223	− 0.0030 (− 0.0051, − 0.0008) 0.007554
Female	− 0.0143 (− 0.0169, − 0.0117) < 0.000001	− 0.0067 (− 0.0090, − 0.0044) < 0.000001	− 0.0038 (− 0.0061, − 0.0015) 0.001372
Stratified by age			
< 60	− 0.0122 (− 0.0146, − 0.0098) < 0.000001	− 0.0125 (− 0.0149, − 0.0101) < 0.000001	− 0.0036 (− 0.0057, − 0.0016) 0.000592
≥ 60	− 0.0032 (− 0.0056, − 0.0009) 0.007595	− 0.0032 (− 0.0056, − 0.0009) 0.007708	0.0002 (− 0.0020, 0.0025) 0.836312
Stratified by race			
Mexican American	− 0.0063 (− 0.0101, − 0.0025) 0.001325	− 0.0040 (− 0.0076, − 0.0005) 0.025166	− 0.0003 (− 0.0037, 0.0031) 0.860094
Non-Hispanic White	− 0.0155 (− 0.0182, − 0.0128) < 0.000001	− 0.0067 (− 0.0093, − 0.0042) < 0.000001	− 0.0047 (− 0.0071, − 0.0022) 0.000163
Non-Hispanic Black	− 0.0074 (− 0.0102, − 0.0046) < 0.000001	− 0.0027 (− 0.0054, − 0.0001) 0.045752	− 0.0011 (− 0.0037, 0.0015) 0.408969
Other Race	− 0.0057 (− 0.0149, 0.0036) 0.230716	0.0005 (− 0.0082, 0.0092) 0.908841	− 0.0037 (− 0.0122, 0.0048) 0.398373

Data are presented as β coefficient (95% CI).

Adjusted covariates: Model 1 = non-adjusted; Model 2 = Age, Sex, Race; Model 3 = Model 2 + PIR, BMI, Total spine BMD, Alkaline phosphatase, Blood urea nitrogen, Total Cholesterol, Total protein, Serum uric acid, Calcium, Creatinine, Phosphorus, HDL-C, LDL-C, CRP, Vit D, Alcohol, Coronary heart disease, Physical activity, Cancer, Smoking.

### Age differences in the relationship between PTH levels and TBS

A linear regression of the age specific association between the PTH level and TBS is shown in Table 4. A significant association was found in young people (age < 60) (β = − 0.0122; 95% CI, − 0.0146, − 0.0098; P < 0.000001). After adjusting for all covariates, negative associations were still noted in young people (β = − 0.0036; 95% CI, − 0.0057, − 0.0016; P < 0.05).

### Race differences in the relationship between serum PTH levels and TBS

A linear regression of the race specific association between the serum PTH level and TBS is shown in Table 4. A significant association was found in Non-Hispanic White (β = − 0.0155; 95% CI, − 0.0182, − 0.0128; P < 0.000001). After adjusting for all covariates, negative associations were still noted in Non-Hispanic White (β = − 0.0047; 95% CI, − 0.0071, − 0.0022; P < 0.05).

### Weighted generalized additive model and a smooth curve fitting

**Figure 2:**
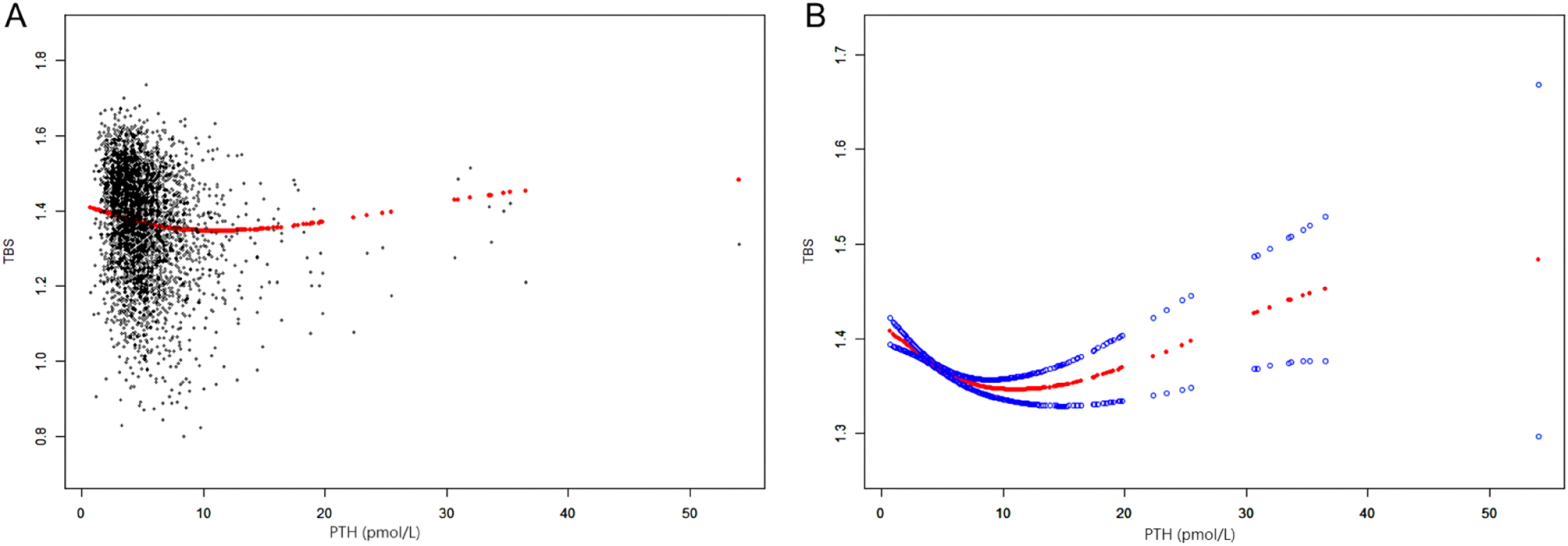
The relationship between the serum PTH and TBS. (**A**) Each black point represents a sample. (**B**) Solid rad line represents the smooth curve fit between variables. Blue bands represent the 95% of confidence interval from the fit. Age, Sex, Race, PIR, BMI, Total spine BMD, Alkaline phosphatase, Blood urea nitrogen, Total Cholesterol, Total protein, Serum uric acid, Calcium, Creatinine, Phosphorus, HDL-C, LDL-C, CRP, Vit D, Alcohol, Coronary heart disease, Physical activity, Cancer, Smoking were adjusted.

**Figure 3:**
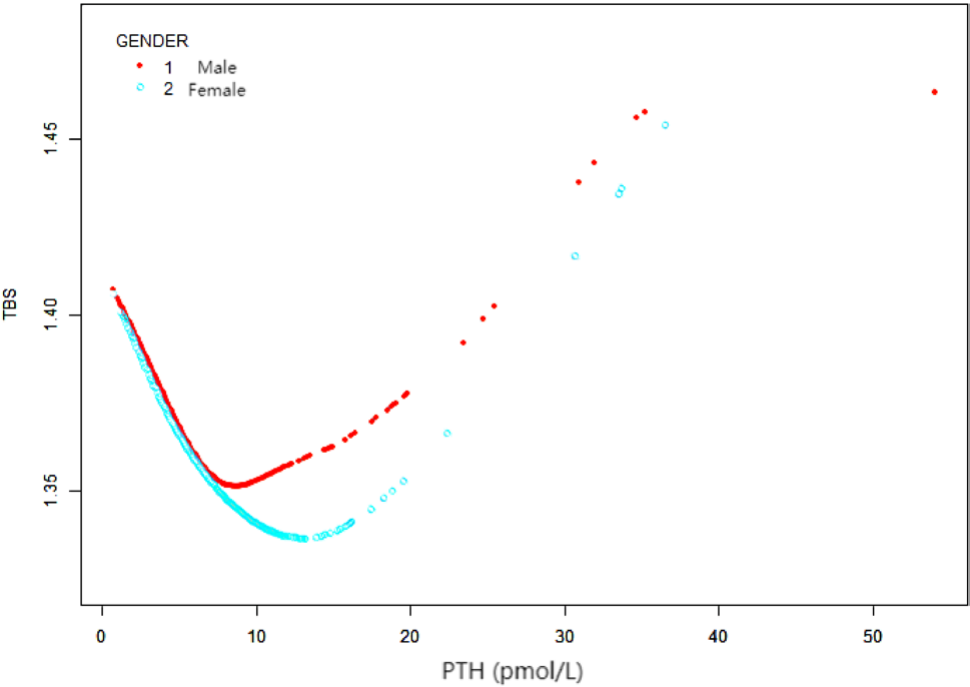
The association between serum PTH and TBS, stratified by gender. Age, Race, PIR, BMI, Total spine BMD, Alkaline phosphatase, Blood urea nitrogen, Total Cholesterol, Total protein, Serum uric acid, Calcium, Creatinine, Phosphorus, HDL-C, LDL-C, CRP, Vit D, Alcohol, Coronary heart disease, Physical activity, Cancer, Smoking were adjusted.

**Figure 4:**
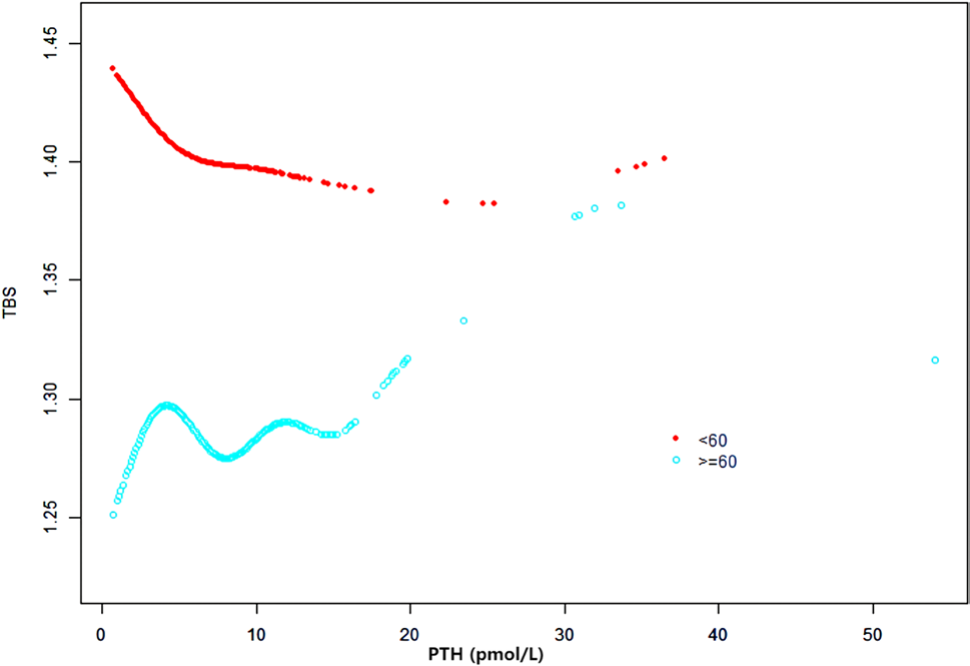
The association between PTH and TBS, stratified by age. Gender, Race, PIR, BMI, Total spine BMD, Alkaline phosphatase, Blood urea nitrogen, Total Cholesterol, Total protein, Serum uric acid, Calcium, Creatinine, Phosphorus, HDL-C, LDL-C, CRP, Vit D, Alcohol, Coronary heart disease, Physical activity, Cancer, Smoking were adjusted.

**Figure 5:**
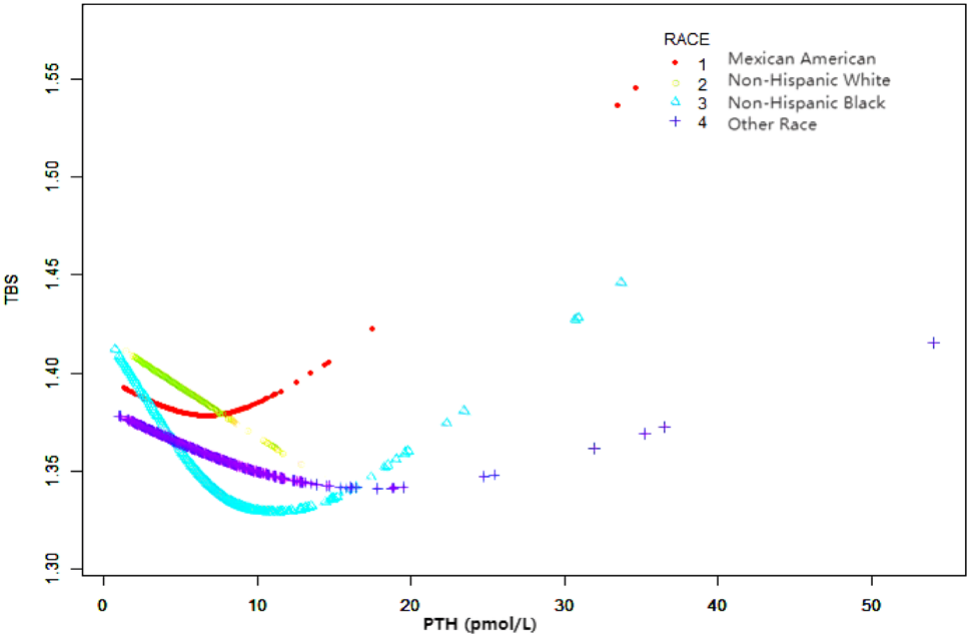
The association between serum PTH and TBS, stratified by ethnicity. Age, Sex, PIR, BMI, Total spine BMD, Alkaline phosphatase, Blood urea nitrogen, Total Cholesterol, Total protein, Serum uric acid, Calcium, Creatinine, Phosphorus, HDL-C, LDL-C, CRP, Vit D, Alcohol, Coronary heart disease, Physical activity, Cancer, Smoking were adjusted.

We used a weighted generalized additive model and a smooth curve fitting to address the non-linear relationship and confirm the results. The association between serum PTH levels and TBS is depicted. Each black point represents a sample. The solid rad line represents the smooth curve fit between variables. Blue bands represent the 95% of confidence interval from the fit. After adjusting for all potential covariates, we found a nonlinear relationship between serum PTH levels and TBS (Fig. 2). The nonlinear relationship showed a U-shape with an inflection point of 6.71 pmol/L (Table 5). For serum PTH levels < 6.71 pmol/L, the effect size of the serum PTH level on TBS was -0.0102 (95% CI, − 0.0130, − 0.0073), and for serum PTH levels > 6.71 pmol/L, the effect size was 0.0022 (95% CI, − 0.0003, 0.0048). In the subgroup analysis stratified by gender, age and race/ethnicity, we found a U-shaped relationship in males, females, adults aged < 60, and non Hispanic white (Figs. 3, 4 and 5). The results of the inflection points are shown in Table 5.

**Table 5:** Threshold effect analysis of serum parathyroid hormone levels on trabecular bone score by using two-piecewise linear regression.

Trabecular bone score	Adjusted ß (95% CI), p-value
Total Serum PTH level	
Fitting by standard linear model	− 0.0034 (− 0.0050, − 0.0017) < 0.0001
Fitting by two-piecewise linear model	
Inflection point	6.71
PTH < 6.71 (pmol/L)	− 0.0102 (− 0.0130, − 0.0073) < 0.0001
PTH > 6.71 (pmol/L)	0.0022 (− 0.0003, 0.0048) 0.0820
Log likelihood ratio	< 0.001
Gender	
Male	
Fitting by standard linear model	− 0.0030 (− 0.0051, − 0.0008) 0.0076
Fitting by two-piecewise linear model	
Inflection point	7.7
PTH < 7.7 (pmol/L)	− 0.0072 (− 0.0106, − 0.0038) < 0.0001
PTH > 7.7 (pmol/L)	0.0017 (− 0.0019, 0.0053) 0.3572
Log likelihood ratio	0.002
Female	
Fitting by standard linear model	− 0.0038 (− 0.0061, − 0.0015) 0.0014
Fitting by two-piecewise linear model	
Inflection point	11.5
PTH < 11.5 (pmol/L)	− 0.0144 (− 0.0195, − 0.0092) < 0.0001
PTH > 11.5 (pmol/L)	0.0011 (− 0.0021, 0.0042) 0.5031
Log likelihood ratio	< 0.0001
Age	
< 60 year	
Fitting by standard linear model	− 0.0036 (− 0.0057, − 0.0016) 0.0006
Fitting by two-piecewise linear model	
Inflection point	5.61
PTH < 5.61 (pmol/L)	− 0.0096 (− 0.0135, − 0.0056) < 0.0001
PTH > 5.61 (pmol/L)	0.0004 (− 0.0027, 0.0034) 0.8087
Log likelihood ratio	< 0.001
≥ 60 year	
Fitting by standard linear model	0.0002 (− 0.0020, 0.0025) 0.8363
Fitting by two-piecewise linear model	
Inflection point	2.64
PTH < 2.64 (pmol/L)	0.0776 (0.0387, 0.1165) < 0.0001
PTH > 2.64 (pmol/L)	− 0.0006 (− 0.0029, 0.0016) 0.5794
Log likelihood ratio	< 0.001
Race	
Mexican American	
Fitting by standard linear model	− 0.0193 (− 0.0553, 0.0167) 0.2932
Fitting by two-piecewise linear model	
Inflection point	7.45
PTH < 7.45 (pmol/L)	− 0.0893 (− 0.1445, − 0.0342) 0.0016
PTH > 7.45 (pmol/L)	0.0632 (0.0021, 0.1243) 0.0429
Log likelihood ratio	< 0.001
Non-Hispanic Black	
Fitting by standard linear model	− 0.0327 (− 0.0562, − 0.0092) 0.0065
Fitting by two-piecewise linear model	
Inflection point	8.75
PTH < 8.75 (pmol/L)	− 0.0905 (− 0.1252, − 0.0558) < 0.0001
PTH > 8.75 (pmol/L)	0.0469 (0.0046, 0.0892) 0.0298
Log likelihood ratio	< 0.001

Data are presented as β coefficient (95% CI).

Adjusted covariates: Model 1 = non-adjusted; Model 2 = Age, Sex, Race; Model 3 = Model 2 + PIR, BMI, Total spine BMD, Alkaline phosphatase, Blood urea nitrogen, Total Cholesterol, Total protein, Serum uric acid, Calcium, Creatinine, Phosphorus, HDL-C, LDL-C, CRP, Vit D, Alcohol, Coronary heart disease, Physical activity, Cancer, Smoking.

Abbreviations: see Table 1.

## Discussion

In this cross-sectional study of American adults, we analyzed the independent effects of serum PTH levels on TBS in 3516 individuals and revealed an independent association between these two variables. We demonstrated a significant negative association between the serum PTH level and TBS in American adults after adjustment for all covariates. Our study suggested that elevated serum PTH levels correlated with decreased TBS, especially in non Hispanic white adults under 60 years old. We also observed a nonlinear U-shaped relationship between serum PTH levels and TBS, with a turning point at 6.71 pmol/L. For a serum PTH < 6.71 pmol/L, every 1 pmol/L increase in serum PTH level was associated with a decrease of 0.0102 in TBS, which was statistically significant. Similarly, this relationship also applies to non-Hispanic white individuals under the age of 60, regardless of gender. Therefore, we can conclude that maintaining PTH levels in a lower reasonable clinical range can help improve TBS indicators and reduce the risk of fractures, thus better preserving bone health.

Parathyroid hormone (PTH) is a hormone produced by the parathyroid glands that regulates calcium and phosphorus metabolism, primarily acting on bones, kidneys, and the intestines^18^. The release of PTH is mainly controlled by Ca^2^^+^ and it also stimulates the kidneys (distal renal units) to reabsorb Ca^2^^+^ (along with magnesium) and inhibits the reabsorption of phosphate^19^. PTH promotes bone formation by encouraging the proliferation of mesenchymal stem cells and inhibiting the release of sclerostin^20,21^. The formation of osteoclasts is regulated by the balance of receptor activator of nuclear factor k-B ligand (RANKL) and osteoprotegerin (OPG) concentrations^22^. PTH can regulate the expression of the RANKL and its soluble decoy receptor OPG in osteoblasts and bone cells, thereby affecting the generation of osteoclasts^23,24^. This effect of bone anabolism is used to treat severe osteoporosis, and is called “anabolic window”. This mechanism of bone formation without absorption stimulation may lead to the increase of newly formed trabecular bone and cortical bone by more than 20%^25,26^. Prior investigations have delineated that PTH is discharged in an oscillatory fashion, characterized by two distinct temporal phases^27^. The first phase, referred to as the homeostatic phase, encompasses a consistent frequency and amplitude of PTH secretions per diurnal cycle, primarily responsible for maintaining equilibrium in bone mass and orchestrating bone metabolism. The second phase, known as the dynamic phase, exhibits a fluctuating kinetics of PTH secretion on a minute-to-minute basis, predominantly serving to regulate serum calcium homeostasis.

Previous studies used high-resolution peripheral quantitative computed tomography (HR-pQCT) to investigate the relationship between bone microarchitecture and PTH level^28,29^. They revealed a association between these two factors. For instance, one study revealed that alterations in PTH secretion patterns were associated with decreased trabecular bone volume and increased cortical porosity in patients with primary hyperparathyroidism^30^. Although HR-pQCT has a higher resolution that allows for more precise detection of bone microarchitecture abnormalities, its application is not yet widespread. TBS is a novel gray-level texture analysis technique based on DXA images, which has been proven to be closely related to direct measurements of bone microarchitecture and the risk of fractures^31^. TBS analysis can easily be conducted from lumbar spine DXA images. Several studies have used a TBS threshold (TBS = 1.200) to identify patients at high risk of fractures^32,33^. Research by Boutroy et al. has shown that women without osteoporosis, but with TBS values below 1.209, have a significantly increased rate of fractures^34^. Another study indicated that approximately 72% of primary hyperparathyroidism (PHPT) patients (TBS < 1.35) exhibit bone microarchitecture deterioration, whereas only 46% were diagnosed with osteoporosis or osteopenia through T-scores^35^. Similarly, recent research by Romagnoli et al. found that mild PHPT patients had lower TBS values compared to age-matched healthy controls, despite having similar lumbar spine BMD measured by DXA^36^. In addition, a cross-sectional study evaluated the relationship between TBS and volumetric BMD, bone microarchitecture, and bone stiffness. The results^37^ showed that TBS was positively correlated with total bone stiffness and other HRpQCT metrics, except for radial trabecular thickness and trabecular stiffness. It was inferred that TBS could reflect bone microstructure and bone strength to some extent and could be a useful tool to prevent osteoporotic fractures.

PTH plays a important role in the regulation of bone metabolism. Its regulation of bone metabolism is a complex process, capable of both stimulating osteoblasts to promote bone formation and activating osteoclasts to regulate bone resorption^38,39^. Studies have shown that the impact of PTH on bone metabolism varies depending on the method of administration, demonstrating a dual regulatory effect where small, intermittent doses promote bone formation, while large, continuous doses enhance bone resorption^16,40,41^. Lindsay et al.^42^ compared the changes in BMD in patients before and after intermittent administration of PTH and found that BMD increased significantly after treatment. The study by Ogita et al.^43^ confirmed through both in vivo and in vitro experiments that intermittent application of PTH can effectively promote the differentiation of preosteoblasts into osteoblasts. At the same time, PTH also significantly improves BMD in men with osteoporosis^44^. Furthermore, there is level I evidence indicating that human PTH significantly increases the BMD of all skeletal sites except the radius and significantly reduces the risk of new vertebral and non-vertebral fractures in postmenopausal women^45^. However, numerous studies have shown that patients with persistently high levels of serum PTH or hyperparathyroidism have reduced bone density and a corresponding increased risk of fracture^46,47^.

An increasing body of evidence shows that PTH is closely related to the development of osteoporosis. However, the direct impact of PTH on BMD remains unclear. In certain animal models, PTH has been observed to have a positive effect on BMD^48,49^. However, an observational study revealed that serum PTH levels are negatively correlated with total hip and femoral BMD^50^. A cross-sectional study involving British women found a negative correlation between PTH levels and overall BMD^51^. Similarly, a prospective study in Korea reported a negative correlation between PTH levels and BMD^52^. Mendelian randomization studies also observed a negative correlation between PTH and BMD, noting that this link strengthens with age^53,54^. Furthermore, it has been found that even among patients with PHPT who maintain normal BMD in the lumbar spine, the incidence of vertebral fractures significantly increases^55^. Therefore, serum PTH levels have important clinical significance for the treatment of osteoporosis and the reduction of fracture risk.

Clinically, it has been observed that patients with PHPT exhibit a correlation between high levels of PTH and reduced bone remodeling and BMD, primarily in the cortical bone^54,56^. Parathyroidectomy (PTX) is beneficial for the skeleton, significantly increasing the percentage of BMD^57^. However, for most patients with fragile fractures, the T-scores usually fall within the low bone mass or even the normal BMD range, failing to accurately reflect fracture risk. In contrast, the TBS serves as a tool to obtain more comprehensive skeletal data, helping to identify individuals with normal BMD but deteriorated microarchitecture^58^. Moreover, the ability of TBS to predict fractures is not affected by BMD and most clinical risk factors^59,60^. The study found that the average TBS values in patients with PHPT were significantly lower than those in the control group^36^. Among patients with PHPT and vertebral fractures, TBS was significantly lower than in patients without fractures. A prospective cohort study conducted in Italy showed a significant increase in TBS within two years after PTX^61^. These findings suggest that higher PTH levels may be associated with poorer bone microarchitecture and higher fracture risk. The results are basically consistent with our study, which proves the reliability of the study results.

Our research indicates a significant negative correlation between PTH levels and total TBS, a finding that remains statistically significant both before and after adjusting for covariates. In subgroup analyses, this negative correlation persists, especially pronounced among non-Hispanic whites under the age of 60. We also observed that, compared to patients under 60, older patients generally have lower TBS values. Additionally, we revealed a U-shaped relationship between PTH levels and TBS, which may correspond to the bidirectional regulatory effects of PTH on bone metabolism. This is particularly evident when serum PTH levels are low (PTH < 6.71 pmol/L), showing a significant statistical difference. Therefore, to promote bone health and further prevent fractures, it is advisable to maintain PTH levels within a relatively low and reasonable range in clinical practice. Notably, in patients with hyperparathyroidism, regardless of the presence of symptoms, early intervention to keep PTH at an appropriate level is recommended to reduce the risk of fractures.

A strength of our study is our use of a representative sample from the general population of the United States and the large sample size. However, the present study has some notable limitations. Firstly, the causal correlations of PTH with TBS were not assessed due to its cross-sectional design. A long-term observation study should be considered in future research. Secondly, we evaluate TBS through DXA, and the operator’s technology may affect the measurement. Thirdly, the study focused on the American population, and it is not clear whether this applies to other regions or countries.

## Conclusion

Our research indicates that among the American population, elevated levels of PTH are associated with decreased TBS. There is a U-shaped curve relationship between the two, with the inflection point at 6.71 pmol/L. Concurrently, this U-shaped curve is also present in non-Hispanic whites under the age of 60, regardless of gender. This finding suggests that maintaining PTH levels as low as possible within the normal range may be more beneficial for bone health and better in preventing fractures.

## Data Availability

The data supporting the results of this study can be obtained from https://wwwn.cdc.gov/nchs/nhanes/Default.aspx. The availability of this data is not restricted.
